# UBA6 and Its Bispecific Pathways for Ubiquitin and FAT10

**DOI:** 10.3390/ijms20092250

**Published:** 2019-05-07

**Authors:** Fengting Wang, Bo Zhao

**Affiliations:** Engineering Research Center of Cell and Therapeutic Antibody, Ministry of Education, and School of Pharmacy, Shanghai Jiao Tong University, Shanghai 200240, China; wft1997@sjtu.edu.cn

**Keywords:** ubiquitin, ubiquitin proteasome system, ubiquitin-like protein, FAT10, UBA6, USE1

## Abstract

Questions have been raised since the discovery of UBA6 and its significant coexistence with UBE1 in the ubiquitin–proteasome system (UPS). The facts that UBA6 has the dedicated E2 enzyme USE1 and the E1–E2 cascade can activate and transfer both ubiquitin and ubiquitin-like protein FAT10 have attracted a great deal of attention to the regulational mechanisms of the UBA6–USE1 cascade and to how FAT10 and ubiquitin differentiate with each other. This review recapitulates the latest advances in UBA6 and its bispecific UBA6–USE1 pathways for both ubiquitin and FAT10. The intricate networks of UBA6 and its interplays with ubiquitin and FAT10 are briefly reviewed, as are their individual and collective functions in diverse physiological conditions.

## 1. Introduction

**Figure 1:**
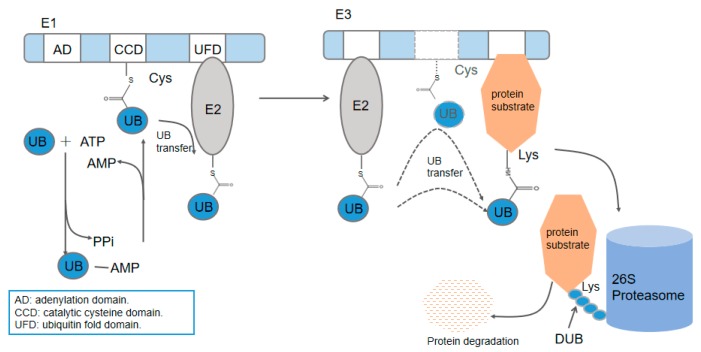
The attachment of ubiquitin (UB) to substrate proteins is carried out through an E1–E2–E3 cascade. UB is directly transferred from E3 to the substrate or from E2 with the mediation of E3. The dotted line indicates two different ways of E3 transfer.

Ubiquitination, the post-translational modulation of protein by ubiquitin (UB), plays an important role in almost all cellular functions in eukaryotes [1,2,3]. The attachment of ubiquitin to substrate proteins is carried out through an E1–E2–E3 cascade transfer. E1, the ubiquitin-activating enzyme, catalyzes ubiquitin C-terminal acyl-adenylation and attacks the ubiquitin adenylate by its active Cys residue to form a thioester bond with ubiquitin in the presence of ATP. E2, the ubiquitin-conjugating enzyme, accepts the ubiquitin and forms the second thioester bond with its active Cys residue. Finally, the ubiquitin is covalently attached to the target proteins via its C terminus to the ε-amino group of the Lys residues in substrates, generally mediated by E3, the ubiquitin ligase [4,5]. After the formation of an isopeptide bond between ubiquitin and its target proteins, a polymeric chain can be synthesized through the linkage of the ubiquitin C terminal to the previous ubiquitin molecule. Ubiquitin consists of seven different Lys sites (K6, K11, K27, K29, K33, K48, K63). Together with their amino terminus Met residue, the eight residues serve as the linkage sites for subsequent ubiquitin molecules to form different types of ubiquitin chains. Mono-ubiquitin modification and polymeric chains elicit different outcomes of the targeting proteins. Predominant K48 homogeneous ubiquitin chains can target the conjugated protein to the 26S proteasome for degradation whereas the second-most abundant chain type K63 ubiquitin chains present nonproteolytic functions in cell signaling [4,6]. Protein can be rescued from degradation by the ubiquitin proteasome system (UPS, and in this review we refer to UPS as an abbreviation for both ubiquitin and the ubiquitin-like protein FAT10 proteasome system) through deubiquitinases (DUBs), which remove the ubiquitin from its substrates before degradation. In line with the intricate ubiquitination cascades, DUBs abolish the ubiquitin signal with equally high complexity [7] (Figure 1). 

Proteins with a B-grasp fold structure similar to that of ubiquitin are referred to as ubiquitin-like proteins (UBLs) [8]. Similar enzymatic cascades that are evolutionarily related to the ubiquitination cascades (E1–E2–E3) catalyze UBLs to their specific substrates. While some of the UBLs have biological functions such as sulfur transfer or lipid modification, a subset of UBLs also participate in protein modification in eukaryotic cells. Ubiquitin-like proteins include small ubiquitin-like modifier 1–3 (SUMO1–3), neural precursor cell expressed, developmentally down-regulated 8 (NEDD8), autophagy-related protein 12 (Atg12), autophagy-related protein 8 (Atg8), interferon-stimulated gene 15 (ISG15), and FAT10 [9]. So far, FAT10 is the only UBL other than ubiquitin that has been described to play an independent role in protein degradation by proteolytic targeting [10]. While most UBLs have their discrete E1, E2, and E3 enzymes, FAT10 shares a common E1 and E2 with ubiquitin, adding complexity about how they differ and coexist with each other in the common pathway [11,12].

Overall, there are eight E1s that have been discovered in humans that initiate UBL conjugation—all of which share a conserved adenylation domain responsible for UBL recognition and acyl-adenylation [5]. Bacterial proteins MoaD and ThiS have a structural homology and functional similarities with eukaryotic UBLs and can be respectively activated to form C-terminal acyl-adenylation by bacterial enzymes MoeB and ThiF, both of which have a sequence homology with eukaryotic E1 adenylation domains [9,13]. In contrast to the at least 40 E2s and 700 E3s that have been identified in the human genome, there are only two E1s, known as ubiquitin-like modifier-activating enzyme 1 (UBE1) and ubiquitin-like modifier-activating enzyme 6 (UBA6), reported to serve as the starting enzyme for the extensive downstream ubiquitination cascades [14,15,16]. Previously, UBE1 was thought to be the sole E1 enzyme that catalyzes the activation of ubiquitin. The discovery of UBA6 in 2007 overthrew the former theory, revealing an unexplored area of the ubiquitin system. UBA6 was found to have all the major architecture of E1 and can charge ubiquitin with the same efficiency as UBE1 [11,14,17]. Besides, UBA6 can also activate the UBL protein FAT10 and transfer FAT10 to its substrate proteins [11]. The FAT10ylation of a target protein may lead to its proteasomal degradation independently from ubiquitin [10]. Although UBA6 and UBE1 share a set of overlapping E2s, both E1s have their dedicated E2s. USE1—the only UBA6-specific E2—can accept ubiquitin and FAT10 only from UBA6, while several E2s are charged solely by UBE1. Intriguingly, FAT10ylation shares a common pathway with ubiquitination through the UBA6–USE1 pathway.

The discovery of UBA6 coexisting with UBE1 and the unique UBA6–USE1 cascade for both FAT10 and ubiquitin put forward interesting questions regarding their individual functions and how they work together. Answers to these questions can facilitate our understanding of the significance of UBA6 in the UPS system. In this review, we give a summary of the research progress on UBA6 and its bispecific pathways for ubiquitin and FAT10 in the past decade.

## 2. UBA6 

The discovery that UBA6 (also known as E1-L2 or UBE1L2) can activate both ubiquitin and FAT10 challenged two old beliefs. First, UBE1 was no longer the only E1 found to activate and transfer ubiquitin to the subsequent proteins. Second, UBA6 became an E1 that could simultaneously activate two very dissimilar ubiquitin-like modifiers (FAT10 shares 29% and 36% identity with ubiquitin at the N-terminus and C-terminus, respectively) [18,19]. UBA6 is 42% identical to UBE1 and 36% identical to the ISG15 E1 enzyme UBE1L [18]. While UBE1 is widely expressed in mammals, yeasts, and plants, UBA6 and its homologs can only be found in vertebrates and sea urchins [14]. As for the question of why two E1s coexist in the higher organism, UBA6 seems to differentiate itself with UBE1 in several aspects. First, although UBA6 and UBE1 share common E2s and E3s for ubiquitin, both E1 enzymes have their dedicated E2s, which can direct ubiquitin towards distinct subsets of E3s and protein substrates. Second, UBE1 is abundant in both the nucleus and cytoplasm, yet UBA6 is more enriched in the cytoplasm [20]. While both E1s are widely expressed in human tissues, UBE1 is over ten times more abundant than UBA6 in several cell lines, indicating its major role in the UPS system [14]. Nevertheless, the expression of UBA6 is up-regulated in certain circumstances, suggesting that UBA6 may exert specific functions or compensate for UBE1 under certain conditions [21,22]. Third, UBA6 and its specific E2 USE1 are the only E1 and E2 enzymes found in the FAT10-proteasome signaling pathway—another branch of the UPS system [12]. All of this evidence points out a non-redundant role of UBA6 in the UPS. Indeed, the deletion of UBA6 in mice caused embryonic lethality, suggesting the irreplaceable role of UBA6 in embryogenesis [11]. Studies in recent years found that UBA6 is associated with several diseases, which may help us understand its novel physiological functions.

### 2.1. UBA6 with Neuronal Diseases

Studies have identified the critical role of UBA6 in brain development. The absence of UBA6 leads to numerous behavior disorders [23,24]. Mice with UBA6-deficient (Uba6^NKO^) brains displayed several pathological phenotypes, such as social interaction deficits, learning and memory defects, and increased metabolic rates. These are believed to at least partly be the effects of morphological defects in the brain. Uba6^NKO^ mice exhibited smaller organismal sizes, reduced numbers of neurons, and decreased dendritic spine densities in the brain. The defects were observed specifically in the CA3 region of the hippocampus and amygdala [24]. An increase in the abundance of HECT (Homologous to E6AP C–terminus)-domain ubiquitin ligase ubiquitin protein ligase E3A (Ube3a) as well as the post-synaptic density protein Shank3 in the amygdala may be related to the observed phenotype. Ube3a is related with Angelman syndrome and autism spectrum disorders (ASDs) [25,26]. The regulation of Ube3a by UBA6 cascades in UPS has been supported by two facts. First, the mRNA levels of Ube3a were not changed, suggesting a post-transcriptional regulation [24]. Second, while Ube3a itself was serving as an E3 ligase, it was self-ubiquitinated by a UBA6 and UBE1 cascade, which controlled its stability in vitro [24,27]. Both catalytic cysteines within USE1 and the HECT domain of Ube3a were required for Ube3a ubiquitination through UBA6–USE1 cascade in which a K48-dependent polyubiquitination was also demonstrated. The Ube3a substrate Arc—an activity-regulated cytoskeleton-associated protein required for persistent forms of synaptic plasticity and memory in the mammalian brain [28]—was subsequently decreased in UBA6-depleted mice [24,28]. However, the observed phenotype may have been an integrated result of multiple UBA6-dependent targets, since other ubiquitinated and FAT10ylated substrates such as regulator of G-protein signaling (RGS) proteins, polyglutamine proteins, and p62 are also related with neuronal development and mental diseases [20,29,30].

As the dysregulations of social interaction and communication are diagnostic symptoms of the autism spectrum, the UBA6^NKO^ mice are suggested to be novel animal models to study ASD. In fact, UBA6′s correlation with neuronal diseases has been found in humans in multiple reported patients. UBA6 gene was found duplicated in patients with attention-deficit hyperactivity disorder and intellectual disorder (ID) or deleted in patients with ID and behavioral disorders [31,32,33,34,35]. It was significantly and strongly expressed in Alzheimer’s disease (AD) human brains compared with normal human brains [36]. The expression of UBA6 was markedly decreased in schizophrenia, which may elicit abnormal changes in several downstream molecular pathways including the decrease of c-jun-N-terminal kinase 1/2 (JNK1/2) phosphorylation [37]. Interestingly, the expression level of UBA6-specific USE1 was up-regulated in patients with frontotemporal dementia, which suggests that the abnormal behaviors of the whole UBA6 cascade are involved in the pathogenesis of neuronal diseases [38].

### 2.2. UBA6 with Cancer Development

UBA6 is vital in the prevention of mammary oncogenesis. UBA6-depleted MCF-10A cells (breast epithelial cells) went through spontaneous epithelial–mesenchymal transition (EMT)—a critical step of mammary cancer development. UBA6 depletion promoted cell proliferation in spite of fully engaged cell–cell contact, the deprivation of growth factors in monolayer culture, or ECM support in 3-D culture [39]. UBA6-specific substrates cytoskeleton linker protein ezrin (EZR) and the Rho-GTPase CDC42 were found to be critical in UBA6-mediated epithelial homeostasis since CDC42 inhibitor ML141 reversed UBA6-deficient cells from the EMT phenotype and the co-expression of anti-EZR and anti-UBA6 shRNAs retrieved lumen formation from 30% of epithelial cells to 70% [39,40]. Both ezrin and CDC42 function in cell morphology and migration, which could influence epithelial morphogenesis in several manners [41,42]. Moreover, both proteins are validated UBA6-specfic targets, indicating the involvement of UBA6 regulation in epithelial biology. Furthermore, MCF-10A cells with a stable UBA6 knockdown displayed an abnormal diffusion of ezrin localization in cytoplasm and nuclei, suggesting a role of UBA6 in ezrin mislocalization [40]. The switch of ezrin localization has been correlated with poor prognosis in breast cancer patients [43]. In fact, among 250 invasive breast cancer tissues examined, 38% of the samples showed a weaker or undetectable expression of UBA6 compared with the normal mammary tissues, further demonstrating UBA6′s association with breast cancer progression [39].

Apart from breast cancer, abnormal expressions in UBA6 and its pairing protein were found in several types of carcinoma. UBA6 was up-regulated in hepatocellular carcinoma (HCC) samples compared with the parent livers, while the expressions of both samples exceeded that of the normal control group. In contrast, FAT10 and ubiquitin were down-regulated in both the HCCs and the adjacent non-tumor tissue compared to the controls [44]. UBA6 was found to be significantly mutated in lung adenocarcinoma through genomics analyses [45,46]. The expression of UBA6 protein was down-regulated in bladder cancer cells as well as in prostate cancer cell lines under hypoxic conditions [47,48]. However, exactly how UBA6 exerts its effect in cancer development needs to be further clarified.

### 2.3. UBA6 with Meiosis Initiation

UBA6 expression was previously found to correlate with meiosis initiation [49]. UBA6 was discovered to be five times more highly expressed in mouse testis compared with any other organs [17]. Microarray analysis of UBA6 expression in murine embryonic ovary and postnatal testis found the expression profile of UBA6 had a high standard correlation (0.9) with that of Stra8—a retinoic-acid-targeting gene that regulates meiotic initiation [50]. The study identified a localization pattern of UBA6 during the mitosis–meiosis transition, where the protein initially existed in somatic and germ cells in the neonatal testes yet became restrained to germ cells as the animal aged [49]. More work should be carried out to demonstrate the concrete role of UBA6 in this process. 

In summary, UBA6 participates in multiple pathogeneses of diseases, predictably owing to its central role in UBA6-dependent post-translational modification. Functions of UBA6 are not limited to the above-mentioned processes. Because of its critical functions in the UPS cascades, the roles of UBA6 are seen repeatedly in the following sections.

## 3. The UBA6–Ubiquitin Cascades

**Figure 2:**
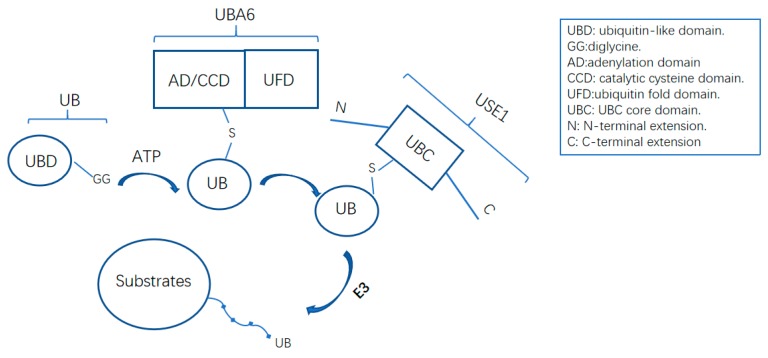
The activation of ubiquitin by UBA6–USE1 cascade. Ubiquitin forms a thioester bond with the catalytic cysteine of UBA6 and is then transferred in a trans-thiolation to the active cysteine of USE1. The N-terminal extension and LB loop in UBA domain are critical for USE1 recognition.

UBA6 initiates ubiquitin transfer through its specialized subset of ubiquitination cascades. Among the 29 UBC4 and UBC5 families of E2 enzymes tested in vitro by Jin et al., 14 E2s were charged by UBE1 but not UBA6, nine E2s were charged by both enzymes, and only one E2, UBE2Z, was found to charge solely with UBA6, and was accordingly renamed as UBA6-specific E2 (USE1) (Figure 2) [14]. The UFD domain responsible for E2 recruitment partly contributes to E1 selectivity toward different E2s since the replacement of UBA6 UFD by UBE1 UFD led to UBA6 charging with UBE1 specific E2 CDC34B. However, the switch of UBE1 UFD with UBA6 UFD did not empower UBE1 to charge with USE1. Interactions outside of UFD in UBA6 may be an explanation. Specific structures in USE1 for mutual selectivity were already identified to explain the insufficiency of UBA6 UFD for E2 recognition [51]. Different E2s interacted with multiple E3s, by which the transfer of ubiquitin to target substrates was directed [52]. Downstream E3s and substrates may overlap in distinct E1–E2–E3 pathways. Together they constitute an extensive and intricate ubiquitination network led by only two E1s. 

### 3.1. Spatial Differences of the UBA6 and UBE1 Cascades

Some UBA6 cascades work in parallel with UBE1 cascades, while they may act in different spaces. The UBA6–USE1 cascade interacts with the UBR1-3 subfamily of N-recognin E3s to regulate the degradation of N-end rule substrates RGS4, RGS5, and Arg-GFP, in parallel with the UBE1–UBE2A/B pathway. However, the USE1-depleted cells mainly exhibited an accumulation of RGS proteins in the cytoplasm, whereas the knockdown of UBE2A/B led to a high level of RGS proteins in both nucleus and cytoplasm. The co-depletion of UBE2 and USE1 resulted in a higher level of RGS accumulation compared with their sole deletions, suggesting that UBA6 and UBE1 functions in a non-overlapping manner [20]. Apart from the RING domain E3s UBR1–3, the founding member of HECT-domain ubiquitin ligases, Ube3a (also known as E6-AP) acts with both E1 cascades as a substrate. Both UBA6–USE1 and UBE1–UBCH7 pathways can direct the ubiquitination of Ube3a, leading to its proteasomal degradation. The former pathway functioned exclusively in cytoplasm whereas the latter functioned in both compartments yet to a lesser extent within the cytoplasm [24].

### 3.2. Identified Ubiquitination Cascades

**Figure 3:**
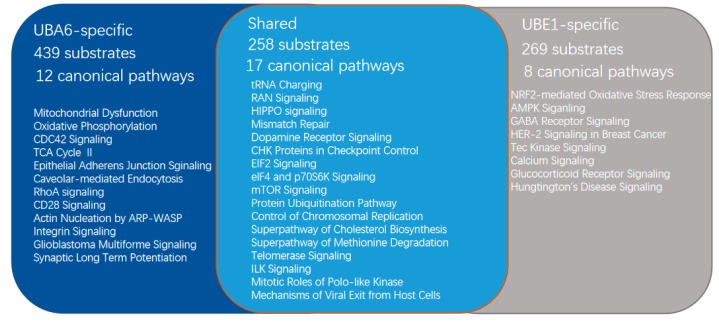
The shared and specific substrates of UBA6 and UBE1 mapped by OUT and Ingenuity Canonical Pathway analyses of the pathways related with the substrates.

The orthogonal ubiquitin transfer (OUT) method proposes a viable approach to mapping the distinctive yet partially overlapping ubiquitination cascades of UBA6 and UBE1 [53]. The method constructed engineered UB and ubiquitination related enzymes (x-UB and X-enzymes) that interacted orthogonally with each other, eliminating cross-reactivity between x-UB and the native enzymes. Protein attached with the tagged x-UB could be identified as a substrate of the corresponding E1 or a specific E3. By this method, Liu et al. succeeded in identifying the specific substrates of UBA6 and UBE1 [40]. In total, they profiled 527 protein substrates for UBA1, 697 for UBA6, and 258 for both in HEK 293 cells (Figure 3). Potential specific substrates of UBA6 include CDC42, CUG triplet repeat binding protein 1 (CUGBP1), and ezrin, and the ubiquitination of these UBA6-specific substrates were verified both in vitro and in vivo. Bioinformatics analysis by Ingenuity Pathway Analysis (IPA) displayed that the x-UB-conjugated substrates identified were associated with multiple cellular pathways, among which 12 pathways had statistically significant associations with UBA6 specific substrates. These pathways included mitochondrial dysfunction, CDC42 signaling, and actin cytoskeleton signaling [40]. The identified substrates and cellular pathways are conducive to the understanding of unique UBA6 functions.

## 4. The UBA6–FAT10 Cascades

Human leukocyte antigen (HLA)-F-adjacent transcript 10 (FAT10) is the only UBL found as a signal for proteasomal degradation by 26S proteasome independent of ubiquitin [10]. FAT10 is a 165-amino acid protein found only in mammals. It is encoded in the MHC locus and is constitutively expressed in mature dendritic cells and B cells [54]. The highest expression of FAT10 mRNA is restrained in the tissues of the immune system, especially the thymus [19]. However, FAT10 is strongly and synergistically inducible in all tissues by treatment with tumor necrosis factor (TNF)–α and pro-inflammatory cytokine interferon (IFN)-γ [55]. During the maturation of human dendritic cells, FAT10, FAT10-specific E1 UBA6, as well as its binding adaptor NEDD8 ultimate buster–1L (NUB1L) were up-regulated under all maturation regimens [22]. This suggests a major function of FAT10 in the immune system. The expression of FAT10 is cell-cycle regulated. The highest expression of FAT10 was observed in the S phase, yet the expression lowered at the G2/M border of the cell-cycle stage [56]. Upon cell release from mitotic arrest, most of the proteins that were FAT10ylated showed a dramatically decreased FAT10 conjugation level in contrast to the significant increase in ubiquitination. The level of USE1 dropped precipitously at the metaphase/anaphase transition, consistent with securin, a FAT10ylation signal. The inhibition of the FAT10 pathway through the knock-down of USE1 or FAT10 led to prolonged mitotic arrest and cell death, which gives strong support that the FAT10 conjugation pathway is involved in mitotic regulation [57].

The involvement of FAT10 is implicated in multiple biological activities, including antigen processing, antimicrobial defense, apoptosis, and oncogenesis [58,59,60]. The over-expression of the FAT10 gene has been observed in several types of cancers, including HCC [61,62,63]. FAT10 is considered to be an oncogene that is associated with cellular malignancy, probably through its interaction with mitotic arrest-deficient 2 (MAD2) [64]. Although mice lacking FAT10 were viable and fertile, FAT10 knock-out mice demonstrated a high level of sensitivity towards endotoxin challenge. Lymphocytes derived from these mice were more prone to spontaneous apoptotic cell death [65]. Nevertheless, FAT10-deficient mice exhibited an extended lifespan, reduced adiposity, and no development of age-associated obesity [66].

### 4.1. The FAT10 Structure

FAT10 was initially called “diubiquitin”, since the FAT10 protein is composed of two ubiquitin-like β-grasp folds at N and C termini, respectively. The two ubiquitin-like domains share 18% identity with each other and are connected through a linker [67,68]. Different surfaces of the two domains render their unique binding specificities. The C-terminal domain interacts with proteasome subunit RPN10 while the N-terminal domain is associated with the adaptor protein NUB1L, which accelerates FAT10 degradation [69,70]. The FAT10 linker was found essential for FAT10 activation and conjugation, since the mutation of the linker abolished the FAT10ylation of specific substrates such as USE1 while the deletion of the linker abrogated FAT10 conjugation [68,71]. The tetrapeptide CYCI in the FAT10 C-terminus affects the selectivity towards UBA6 and USE1. The replacement of the C-terminal tetrapeptide from CYCI to LRLR, which contributes to the selectivity of ubiquitin and ISG15 for their cognate E1s, resulted in the loss of specificity of FAT10 loading to the E1 and E2 enzymes. Furthermore, the CYCI tetrapeptide in FAT10 lowered its transfer rate from E1 to E2, since the FAT10 variant with this motif replacement by the LRLR displayed an increased rate of transfer to USE1 compared with the wild-type FAT10 [51].

### 4.2. Regulation of the FAT10 Cascade

Since its discovery in 2007, UBA6 has been found to activate FAT10 in the presence of ubiquitin. The thioester bond can be formed between UBA6 and His6-FAT10 instead of a larger GST-tagged FAT10 in vitro [11,14]. The knockdown of UBA6 with siRNA prevented the formation of FAT10 conjugates, indicating that UBA6 is both necessary and sufficient to activate FAT10 [11]. By the same approach, the silencing of USE1 led to a significant reduction of FAT10 conjugates under endogenous and over-expression conditions, suggesting that USE1 is the major, if not the only, E2 enzyme in the FAT10 cascade [12]. Interestingly, USE1 undergoes self-FAT10ylation in *cis*. The self-FAT10ylation of USE1 did not abrogate the activity of USE1 since the free active site cysteine of the USE1–FAT10 conjugate was still loaded with activated ubiquitin or other FAT10. However, the FAT10ylation of USE1 accelerated its self-degradation. Therefore, there seems to be a negative feedback regulation of the FAT10ylation cascade at E2 level, where the self-FAT10ylation of USE1 limits the FAT10ylation pathway and may also influence the ubiquitination cascade through UBA6 [71].

So far, no FAT10 deconjugating enzyme has been discovered, despite many efforts [10]. It seems that FAT10 is degraded along with its substrates at the proteasome, since the endogenous conjugated FAT10 is as short-lived as the unconjugated FAT10 monomer [10,68]. As an interferon-inducible protein, NUB1 serves as a linker with both VWA domains of proteasome RPN10 and FAT10, thereby forming a ternary complex [69,70]. NUB1 was found to bind with FAT10 non-covalently and significantly accelerated FAT10 degradation by proteasome [72,73]. The replacement of four Cys residues in the ubiquitin fold domains of FAT10 strongly decreased the degradation rate, indicating that the intrinsic instability of FAT10 enables the rapid joint degradation of FAT10 and its substrates [68]. The Leber-congenital-amaurosis-associated aryl hydrocarbon receptor interacting protein-like 1 (AIPL1) can antagonize the NUB1-mediated degradation of the FAT10 conjugate [74,75]. Interestingly, AIPL1 also co-immunoprecipitates UBA6, suggesting that AIPL1 may mediate the FAT10 conjugation machinery directly through UBA6 [74]. The regulation of the FAT10 cascade at the UBA6 level was validated by the LIM domain only 2 protein (LMO2). LMO2 interacted with UBA6 at the ubiquitin fold domain, therefore blocking USE1 recruitment by UBA6. This disturbance led to a decrease of the overall cellular FAT10ylation level, which subsequently resulted in a declining degradation of p62, a proven FAT10 substrate [76].

## 5. Candidates of FAT10ylation Cascades

A proteomic analysis of endogenous FAT10 identified 571 FAT10-interacting proteins under stimulation with TNF-α and IFN-γ in Hela cells [77]; 176 proteins were assigned as putative substrates of FAT10ylation (including 10 E3 ligases), and the remaining 395 proteins were putative non-covalently bound interaction partners [77]. In another study, 175 proteins were identified with high confidence as FAT10ylated candidates, which involved a broad spectrum of cellular processes [78]. Nevertheless, the functional roles of the FAT10ylation of these proteins need further study. Still, a group of FAT10 targets were profiled in a cell-cycle interaction network related with cell-cycle regulation and mitotic progression [57]. So far, no E3 enzyme has been found in the Fat10ylation cascade. Several E3 ligases for ubiquitination or SUMOylation were detected among the candidate pool of FAT10ylation, yet it remains to be determined whether these E3 ligases are substrates for FAT10ylation or the functional E3s in the FAT10ylation cascade [77,78].

Studies have found that FAT10 leads to the proteasomal degradation of p62. The respective downregulation of UBA6 and USE1 resulted in a strong reduction in the amount of endogenous p62–FAT10 conjugates, suggesting that p62 is a FAT10ylation target through the UBA6–USE1 cascade. However, a huge amount of non-covalent interacting p62 was also detected despite the knockdown of UBA6 and USE1, indicating that p62 may interact with FAT10 for non-degradational functions [77]. Conjugate and non-conjugate forms of FAT10 with its substrates may bring about different, even contradictory, results. The covalent FAT10ylation of OTU deubiquitinase, ubiquitin aldehyde binding 1 (OTUB1) led to OUTBI proteasomal degradation whereas a non-covalent interaction stabilized OTUB1 [79]. The FAT10ylation of Wnt-induced secreted protein-1 (WISP1) facilitated its protein degradation. However, the over-expression of FAT10 resulted in a disorder of WISP1 mRNA and protein levels, with the expression of WISP1 protein decreasing and the mRNA expression of WISP1 increasing [80]. This study explained that FAT10 exerted its stabilization and degradation functions simultaneously by promoting WISP1 mRNA expression via stabilizing β-catenin yet directly targeting the WISP1 protein for degradation [80]. Nevertheless, there are converse results of FAT10ylation with tumor suppressor p53, whose reasoning remains unknown. One study found that FAT10 modified p53 and upregulated its transcriptional activity, yet other study reported that the over-expression of FAT10 suppressed the transcriptional activity of p53 [81,82]. To add more complexity, p53 negatively regulated FAT10 expression, presumably through the regulation of proteasome, since a 26S proteasome inhibitor abrogated the inhibitory effect [83].

Previous studies demonstrated that both USE1 and UBE1 were protein substrates of FAT10ylation through the UBA6–USE1 cascade [12,84]. UBE1 and FAT10 formed a non-reducible conjugate under the conditions of the over-expression of FAT10 or the induction of endogenous FAT10 expression in the presence of proinflammatory cytokines. The mono-FAT10ylation of UBE1 led to UBE1 degradation, which was dependent on UBA6 and USE1 [84]. The activation of FAT10 by UBA6 was found sufficient to transfer FAT10 onto UBE1 in vitro. However, the question remains as to whether it was because the high concentration of FAT10 obviated the need of the E2 and E3 enzymes. Besides, only a small portion of UBE1 was found to be modified, adding challenges towards the functional study of UBE1 FAT10ylation. Nevertheless, the findings imply a putative regulatory role of the FAT10ylation cascade on the ubiquitin conjugation pathway via the UBE1 cascade [84].

### Does FAT10 Facilitate Protein Degradation or Not?

Kalveram et al. found that during proteasome impairment, FAT10 interacted with histone deacetylase 6 (HDAC6)—a protein mediating the transport of polyubiquitinated and FAT10ylated proteins towards aggresome [85,86]. FAT10 may associate with HDAC6 to ensure the subsequent degradation of proteins via autophagy, as the author suggested. The interaction only occurred when the FAT10-mediated proteasomal degradation failed to function [85]. However, FAT10 itself may be a reason for the reduced activity of proteasomal degradation. FAT10 is critical for the switch of the 26S proteasomes to immunoproteasomes. This switch causes the formation of Mallory–Denk bodies (MDBs), the aggresome of undigested ubiquitinated proteins [30,87]. FAT10 KO mice failed to form MDBs in the DDC mouse model, as the 26S proteasome could retain normal proteolytic activity without the presence of the FAT10 promoter region, which signals the upregulation of the catalytic subunits of the immunoproteasome [87]. The study identified an upregulation of FAT10 in liver samples from human patients with alcoholic hepatitis, yet downregulated expressions of UBA6 and USE1 [87]. Efforts have been made to prevent the formation of MDBs and preserve the 26S proteasome function [88,89]. The pathogenesis of hepatitis may be a consequence of failure in multiple protein quality control pathways including the FAT10ylation cascade [90].

## 6. Comparison and Interplay between FAT10 and Ubiquitin

### 6.1. Comparison

**Figure 4:**
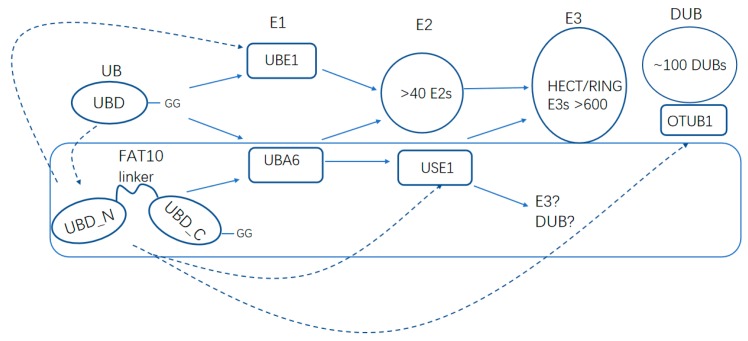
The enzyme cascades for FAT10 and ubiquitin. FAT10 and ubiquitin have mutual influence on the regulations. The dotted lines indicate the regulations of critical enzymes in the cascades by FAT10 and ubiquitin.

While ubiquitin is ubiquitously expressed in all tissues, FAT10 is mostly restricted to the immune system [10]. The C terminus of FAT10 ends with a free diglycine available for instant activation and conjugation, yet ubiquitin and most UBLs need to be processed by protease to a mature form. Mono–FAT10 modification is sufficient for the signaling of proteasomal degradation [77,84], while ubiquitination requires a ubiquitin chain of at least four ubiquitin molecules for efficient proteasomal targeting [19,91]. Unlike ubiquitin, which is recycled by DUBs before the degradation of the substrates, FAT10 is degraded along with its substrates. Usually, most of the FAT10 molecules are degraded within three hours, but ubiquitin is rather long-lived, with an approximate half-life of nine hours [19,71,92]. Furthermore, the ubiquitin-targeted degradation of tightly folded proteins requires ATPase VCP/p97-mediated unfolding. However, the unstructured N-terminal tail of FAT10 allows it to bypass the requirement [68,93]. Other differences lie in the cascades and substrates of FAT10ylation and ubiquitination. In spite of a replete pool of E2 enzymes available for ubiquitination, USE1 is the only demonstrated E2 for FAT10ylation (Figure 4) [15]. Putative pools of ubiquitin and FAT10 seldom overlap, suggesting distinct regulatory functions of the two cascades. While both ubiquitin and FAT10 cooperate with UBA6, FAT10 binds UBA6 with a significantly higher affinity [94]. However, its lower catalytic activity in adenylation and trans-thiolation reaction renders it less efficient for further transfer [94]. Cellular analysis showed that the ratio of free ubiquitin to free FAT10 available for UBA6 decreased significantly under stimulated conditions, while the percentage of thioester-incorporated FAT10 with the total amount of FAT10 dramatically increased, yet that of ubiquitin remained unchanged. It seems that UBA6 becomes specific for FAT10 under physiological changes (e.g., upon stimulation by TNF-α and IFN-γ) [94].

### 6.2. Interplay

Interestingly, ubiquitin was found able to target FAT10. The ubiquitination of FAT10 seemed to accelerate FAT10-mediated degradation, suggesting a direct regulation of ubiquitination on FAT10 [95]. Nevertheless, FAT10 could conjugate with UBE1 and lead it to degradation, potentially exerting an upstream regulatory role of FAT10 on ubiquitin. Furthermore, FAT10 directly interacted with deubiquitinase OTUB1, which stimulated its activity towards K48-linked deubiquitination. FAT10 also led to an increased interaction between OTUB1 and its cognate E2 UbcH5B, which could prevent ubiquitin transfer. The research implied a major inhibitory influence of FAT10 on the ubiquitination processes [79,96]. In fact, FAT10 has been reported to stabilize proteins via antagonizing their ubiquitination processes in several cases. FAT10 stabilized caveolin-3 expression by inhibiting ubiquitination-mediated degradation in cardiomyocytes, therefore inhibiting hypoxia-induced cardiomyocyte apoptosis [97]. FAT10 directly bonded to the DNA-binding transcriptional repressor zinc finger E-box-binding homeobox 2 (ZEB2) and decreased its ubiquitination in breast cancer cells, thereby enhancing its pro-metastasic effect [98]. One possible mechanism for the FAT10 suppression of ubiquitination is that it can compete with ubiquitin for binding to some substrates. For example, FAT10 stabilized eukaryotic translation elongation factor 1A1 (eEF1A1) by competing with ubiquitin for attachment on the same lysines. The over-expression of FAT10 resulted in a decreased UB–eEF1A1 level but an increased FAT10–eEF1A1 level, which contributed to cancer cell development [99].

## 7. USE1

The ubiquitin conjugating enzyme USE1 (also known as UBE2Z) is the only known E2 to accept and transfer ubiquitin solely from UBA6 [12]. The USE1 gene is located on human chromosome 17q21.32 and is widely expressed in human tissues, especially in the placenta, pancreas, spleen, and testis [100]. It is a 246-amino-acid protein with a highly conserved UBC domain found in all E2s, whereas its N- and C-terminal extensions and UBC domain classify it as a class-IV E2 enzyme [51,101]. Western blotting and immunofluorescence staining have proved that USE1 is highly enriched in the cytoplasm, whereas it can shuttle to the nucleus [74]. Structural analysis of USE1 has shown that the N-terminal extension and LB loop of USE1 are critical for USE1 selectivity towards UBA6, since USE1 variants USE1_ΔLB_ and USE1_ΔNter_ lose the ability to bind with ubiquitin-loaded UBA6 [51]. However, the absence of N-terminal extension or the LB loop did not adversely affect USE1 interaction with FAT10-loaded UBA6.

### 7.1. USE1 with Cardiovascular Diseases

Genome-wide association studies (GWASs) have found USE1/rs46522 with regulatory functions in coronary artery disease (CAD) [102,103,104]. USE1/rs46522 is located in the *USE1-GIP-ADTP5G* gene cluster and was associated with CAD in Han Chinese and Iranian populations [105,106]. The regulatory mechanism is not clear, but may involve the linkage disequilibrium of rs46522 with causal single-nucleotide polymorphisms (SNPs) in the gastric inhibitory peptide (GIP) gene, which potentially mediates known CAD risk factors [105]. USE1 mRNA was differentially expressed in myocardial infarction (MI) [107]. Other studies also identified different SNPs located in USE1 with a predicted association with CAD and MI [108,109]. USE1 has also been found related to chronic kidney disease, hyper-triglyceridemia, and type 2 diabetes [110,111]. All of these indicate that USE1 is a susceptible gene for cardiovascular diseases.

### 7.2. USE1 with Human Lung Cancer

Kim et al. found that 92.5% of tumor-normal-paired samples derived from 106 cancer patients showed a significantly upregulated USE1 expression, while the mRNA level of USE1 remained unchanged [112]. The unchanged mRNA level suggests an increased translation or decreased degradation of the USE1 protein in cancer tissues [113]. The overexpression of USE1 promoted the proliferation, migration, and invasion of lung cancer cell lines, whereas the knockdown of USE1 markedly reduced these phenomena. The effect was further confirmed in a xenograft experiment in nude mice. The FAT10–UBA6–USE1 cascade was not likely to be involved in the USE1 overexpression in these samples because no significant difference was observed in the protein or mRNA levels of FAT10. Interestingly, the study also demonstrated the interaction between the anaphase-promoting complex/cyclosome (APC/C) and USE1 and indicated that USE1 was degraded by the E3 ligases APC/Ccdh1 and APC/Ccdc20. Missense mutations in the D-box region of USE1 prolonged the stability of the USE1 protein by compromising its interaction with the APC/C, which was found in 13.2% of cases. It remains unclear how USE1 exerts its functions in cancer cell lines. The N-end rule substrates of the UBA6–USE1 cascade may provide one explanation, since RGS4 and RGS5 are all tumor-suppressor proteins and also are involved in lung cancer [114,115]. However, more evidence needs to be found to elucidate whether the overexpression of USE1 exerts its function by modulating proteostasis in lung cancer cells.

## 8. Concluding Remarks

This review gives a brief summary of the latest research on UBA6 and its two cascades in the UPS system as well the interplay between FAT10ylation and ubiquitination. UBA6 is involved in brain development, cancer progression, and various physiological processes. The abnormal behaviors of proteins in UBA6 cascades are all contributory factors to the altered homeostasis and pathologies. These proteins include substrates as well as constitutive and regulatory enzymes, most of which are promising therapeutic targets for diseases—especially in cancer and neurodegenerative diseases [116,117]. The new layer of bidirectional regulation draws close the relationship between FAT10 and ubiquitin, and raises more curiosity about how their antagonistic yet synergistic functions work. In addition, the identification of the downstream E3s of UBA6-USE1 for both cascades remains challenging. More information on how UBA6 regulates the downstream functions in their relevant cascades is required. Mechanistic studies on their regulation in certain conditions will also help us to better understand the pathogenesis of diseases and provide information for potential drug targets.

## Abbreviations

AD	Alzheimer’s disease
AIPL1	Aryl hydrocarbon receptor interacting protein-like 1
APC/C	Anaphase-promoting complex/cyclosome
ASD	Autism spectrum disorder
Atg8	Autophagy-related protein 8
Atg12	Autophagy related protein 12
CAD	Coronary artery disease
CUGBP1	CUG triplet repeat binding protein 1
DUB	Deubiquitinase
eEF1A1	eukaryotic translation elongation factor 1A1
EMT	Epithelial–mesenchymal transition
EZR	Ezrin
FAT10	Human leukocyte antigen (HLA)-F-adjacent transcript 10
GIP	Gastric inhibitory peptide
GWAS	Genome-wide association studies
HCC	Hepatocellular carcinoma
HDAC6	Histone deacetylase 6
HECT	Homologous to E6AP C terminus
ID	Intellectual disorder
ISG15	Interferon-stimulated gene 15
JNK1/2	c-jun-N-terminal kinase 1/2
LMO2	LIM domain only 2
MAD2	Mitotic arrest-deficient 2
MDB	Mallory–Denk body
MI	Myocardial infarction
NEDD8	Neural precursor cell expressed, developmentally down-regulated 8
NUB1L	NEDD8 ultimate buster-1L
OTUB1	OTU deubiquitinase, ubiquitin aldehyde binding 1
OUT	Orthogonal ubiquitin transfer
RGS	Regulator of G-protein signaling
SUMO1-3	Small ubiquitin-like modifiers 1–3
UB	Ubiquitin
UBA6	Ubiquitin-like modifier-activating enzyme 6
UBC	Ubiquitin conjugating enzyme; UBC core domain
UBE1	Ubiquitin-like modifier-activating enzyme 1
Ube3a	Ubiquitin protein ligase E3A
UBL	Ubiquitin like protein
UPS	Ubiquitin-proteasome system
USE1	UBA6 specific E2
WISP1	Wnt-induced secreted protein-1
ZEB2	Zinc finger E-box–binding homeobox 2
